# Horse Serum in Treatment of Wounds

**Published:** 1916-10

**Authors:** 


**Horse Serum in Treatment of Wounds.** J. Lingnieres, Acad. de Med., Nov. 9, 1915. 10-12 liters of blood can be withdrawn from a horse, every 15-20 days, in two operations. 1/2% of phenol is added immediately after separation from the clot. It is applied on compresses, renewed once or twice a day.

